# Three-year outcome of local injection of autologous stromal vascular fraction cells and microfat in refractory perianal fistulas of Crohn’s disease

**DOI:** 10.1186/s13287-022-02738-x

**Published:** 2022-02-09

**Authors:** Lucas Guillo, Fanny Grimaud, Fanny Houser, Caroline Prost, Elisabeth Jouve, Cécile Philandrianos, Maxime Abellan, Julie Veran, Carine Visee, Laura Beyer-Berjot, Ariadne Desjeux, Françoise Dignat-George, Marc Leone, Jean-Charles Grimaud, Florence Sabatier, Mélanie Serrero, Jérémy Magalon

**Affiliations:** 1 Gastroenterology Department, Assistance Publique Hôpitaux de Marseille, University Hospital of Marseille Nord (AP-HM), Chemin des Bourrely, 13015 Marseille, France; 2 Cell Therapy Department, INSERM CBT-1409, Assistance Publique Hôpitaux de Marseille, Aix-Marseille University, Marseille, France; 3 Department of Radiology, Assistance Publique Hôpitaux de Marseille, Marseille, France; 4 Statistical Department, Assistance Publique Hôpitaux de Marseille, Marseille, France; 5 Plastic Surgery Department, Assistance Publique Hôpitaux de Marseille, Marseille, France; 6 Digestive Surgery Department, Assistance Publique Hôpitaux de Marseille, Marseille, France; 7 C2VN, INSERM UMR 1263, Faculté de Pharmacie de Marseille, Aix Marseille University, Marseille, France; 8 Department of Anaesthesia and Intensive Care, Assistance Publique Hôpitaux de Marseille, Marseille, France; 9 Centre d’Investigation Clinique 1409, Assistance Publique Hôpitaux de Marseille, Aix Marseille University, Marseille, France

**Keywords:** Stromal vascular fraction, Crohn’s disease, Perianal fistulas, Long-term outcome

## Abstract

Perianal fistulas in Crohn's disease are frequent and disabling, with a major impact on patients' quality of life. Cell-based therapy using mesenchymal stem cells represents new hope for these patients, but long-term efficacy remains challenging. In a pilot study, including patients with refractory complex perianal fistulas, autologous adipose-derived stromal vascular fraction (ADSVF) combined with microfat achieved combined remission in 60% of cases, with a good safety profile at 1 year. The purpose of this study is to assess whether these results were maintained at longer term. The safety and efficacy data of the ten patients were evaluated retrospectively 3 years after injection on the basis of clinical and radiological data. MRI were analysed according to the MAGNIFI-CD score. No adverse event was attributed to the experimental stem-cell treatment. Combined remission was achieved in 7 patients (70%) and associated with a significant improvement in the MAGNIFI-CD MRI score. In conclusion, the safety and efficacy of ADSVF and microfat injection in Crohn's disease fistulas were maintained at 3 years, demonstrating that this innovative strategy is effective in producing a long-lasting healing effect. The ongoing multicentre randomized placebo-controlled trial (NCT04010526) will be helpful to define the place for this approach in the current therapeutic arsenal.

## Introduction

Perianal fistulas in Crohn's disease are frequent and disabling, with a major impact on patients' quality of life [1]. Despite optimal treatment, combining biologics and surgical intervention, only 35 to 50% of patients with complex fistulas achieve long-term remission [2, 3]. To address higher remission rate, new strategies are developed, including stem cell therapy. Panes and colleagues evaluated allogeneic mesenchymal stem cells (MSCs) derived from adipose tissue [4, 5] and obtained a 1-year combined remission of 56% (vs 40% in the control group). However, long-term efficacy remains challenging.

Autologous adipose-derived stromal vascular fraction (ADSVF) represents an alternative to ex vivo-cultured MSCs and can be easily obtained within few hours after lipoaspiration. ADSVF corresponds to the cell pellet obtained after enzymatic digestion and removal of adipocytes. This heterogeneous mixture of cells contains not only MSCs, but also immune cells and endothelial cell progenitors that synergistically provide pro-angiogenic, regenerative and immunomodulatory activities [6, 7]. We obtained encouraging results in the ADICROHN pilot study, using local injection of ADSVF associated with microfat: combined remission (defined as the complete cessation of fistula suppuration, with no collection greater than 2 cm on magnetic resonance imaging (MRI)) in six of ten patients (60%) at week 48, with significant improvement in quality of life and a good safety profile [8]. We aimed to assess whether the safety and efficacy obtained 1 year after the local injection of ADSVF and microfat on Crohn’s complex and refractory perianal fistulas could be maintained 3 years after.

## Methods

The ten patients treated in the ADICROHN trial [8] were evaluated at 3 years using clinical examination, the Perianal Crohn's Disease Activity Index (PDAI), the Short Inflammatory Bowel Disease Questionnaire (SIBDQ) and MRI. Safety data were retrospectively collected using the computerized patient record. Eligible patients to the ADICROHN prospective pilot study (NCT 02520843) presented Crohn's disease with low luminal activity (Crohn Disease Activity Index < 250) complicated by one or more complex perianal fistulas refractory to surgical drainage with setons and anti-TNF alpha treatment. Patients were excluded if they had a collection of more than 2 cm, active proctitis, anorectal stenosis, or surgical treatment other than surgical drainage or seton placement.

The cell therapy procedure required one surgical procedure for fat harvesting and another surgical procedure for microfat and ADSVF injection. The fat was harvesting was previously described [9]. ADSVF was obtained within 4 h of lipoaspiration by an automated process (Celution800/CRS system, Cytori Therapeutics, San Diego, USA). Then, ADSVF was aseptically recovered and suspended in 5 mL Lactate Ringer’s solution. The final cell suspension was transferred into a syringe (10 mL) for injection. The treatment administration was also previously reported [8]. A median of 22.8 × 10^6^ [10.9–47.8 × 10^6^] viable nucleated cells was injected, and the median quantity of microfat injected was 10.8 mL [5–17 mL].

Categorical variables were described as percentage, while continuous variables were reported as mean and standard deviation.

## Results

The characteristics and treatments of patients at baseline are available in Table 1. No serious adverse event was reported related to the fat harvesting procedure. Moderate pain at the lipoaspiration site that resolved in less than one week under simple oral analgesia was observed in 4 patients (40%), and one cutaneous reaction secondary to anesthetics was also noticed. The adverse and intercurrent events between the first and the third years of follow-up were recorded and are listed in Table 2. A new fistula tract, in a site other than the treated fistula, appeared in one patient and was treated with fistulotomy. Perianal abscesses occurred in three patients and necessitated draining with setons, and two of them were treated punctually with a combination of antibiotics. Two patients required one or more changes in the maintenance treatment. Finally, two patients received luminal surgeries: one ileocecal resection for stenosis and a new colostomy due to anastomotic leakage after stoma reversal surgery which was permitted by complete healing of fistula at 1 year. No neoplastic changes/transformation was observed. These adverse events were not considered to be related to the cell-based treatment, but rather to the classic and frequent progression of fistulizing perianal disease.

**Table 1:** Patients and disease characteristics at baseline

Characteristics at baseline	*n* = 10
Men	6 (60%)
Age (year)	37.3 ± 12.8
*Smoking status*	
Active smoker	3 (30%)
Non-smoker	3 (30%)
Former smoker	4 (40%)
BMI^†^	24.4 ± 5
CD^‡^ duration (year)	11.8 ± 11.4
CDAI^§^ score	139 ± 116
SIBDQ^††^ score	43 ± 12
Fistula duration (year)	5.2 ± 4.9
PDAI^‡‡^ score	7.3 ± 2.7
*Number of fistula tract*	
1	4 (40%)
2	5 (50%)
> 2	1 (10%)
*Type of fistula*	
Intersphincteric	1 (10%)
Trans-sphincteric	8 (80%)
Suprasphincteric	0 (0%)
Extrasphincteric	1 (10%)
History of fistula surgery	10 (100%)
*Prior fistula treatment failure*	
At least 1 anti-TNFα^§§^ (± IS^†††^)	10 (100%)
2 anti-TNFα	8 (80%)
Vedolizumab	3 (30%)
Derivation ileostomy	2 (20%)
*Baseline treatment*	
Anti-TNFα	7 (70%)
Vedolizumab	3 (30%)

Data represent frequency n (%) or mean and standard deviation

^†^ Body Mass Index

^‡^ Crohn’s Disease

^§^ Crohn’s Disease Activity Index

^††^ Short Inflammatory Bowel Disease Questionnaire

^‡‡^ Perianal Crohn's Disease Activity Index

^§§^ Anti-Tumour Necrosis Factor alpha

^†††^ Immunosuppressant

**Table 2:** Adverse and intercurrent events at 3 years

Events	*n* (%)
Perianal pain	4 (40%)
Local inflammation	4 (40%)
New fistula tract	1 (10%)
*Abscess*	3 (30%)
1	2 (20%)
> 1	1 (10%)
Luminal surgery	2 (20%)
*Anal surgery*	4 (40%)
Drainage with setons	3 (30%)
Fistulotomy	1 (10%)
Change of biotherapy	2 (20%)
*Hospitalization*	4 (40%)
1	2 (20%)
> 1	2 (20%)
Antibiotic therapy	2 (20%)
Steroids	0 (0%)

**Fig. 1:**
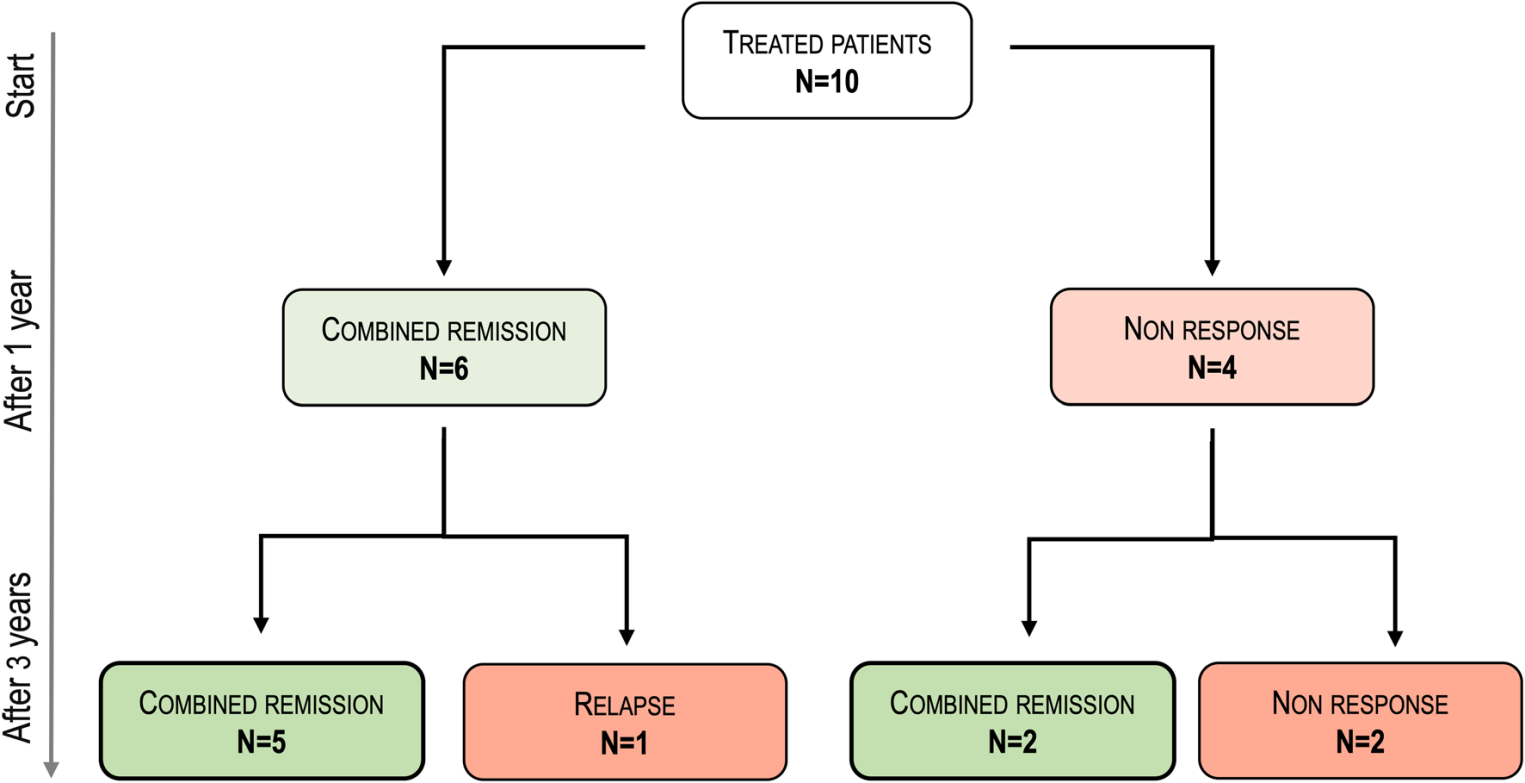
Efficacy results at 1 year and 3 years

At 3 years, 70% of patients had achieved combined remission (Table 3). Five out of six patients (83%) were in combined remission at both 1 and 3 years. One patient in combined remission at 1 year relapsed but did not require a change in therapy or surgical management. Two patients achieved delayed combined remission at 3 years. Finally, two patients were non-responders at one or 3 years and were considered to have failed (Fig. 1). One of the patients who was in combined remission at 1 year and 3 years discontinued maintenance treatment due to an improvement in luminal disease. The baseline and 3-year mean PDAI scores were similar, while they improved at 1 year (Table 3). The significant improvement in quality of life assessed by the SIBDQ score observed at 1 year was maintained at 3 years (Table 3).

**Table 3:** Study outcomes over time

Study outcomes	Baseline	1 year	3 years
Combined remission		6 (60%)	7 (60%)
Response*		2 (20%)	0 (0%)
No response		2 (20%)	3 (30%)
PDAI score^†^	7.3 ± 2.7	3.4 ± 4.3	7.2 ± 5.2
SIBDQ score^‡^	43.4 ± 11.9	47.0 ± 11.4	49.0 ± 13.2

MAGNIFI-CD score	Baseline	1 year	3 years
*All patients (n = 10)*			
IRM total score	17.8 ± 4.9	16.2 ± 6.1	14.7 ± 5.7
Change from baseline	− 1.6 ± 3.1	− 3.1 ± 3.3	
*Patients in remission at 3 years (n = 7)*			
IRM total score	16.8 ± 5.9	14.3 ± 6.0	11.5 ± 4.3
Change from baseline	−2.5 ± 2.4	−5.3 ± 1.8	
*Patients without response at 3 years (n = 3)*			
IRM total score	19.3 ± 3.0	19.0 ± 5.8	19.5 ± 4.0
Change from baseline	0.3 ± 3.9	0.3 ± 1.3	

Data represent frequency *n* (%) or mean and standard deviation

^†^Perianal Crohn's Disease Activity Index

^‡^Short Inflammatory Bowel Disease Questionnaire

^*^Clinical improvement in suppuration but without reach combined remission outcome

MRI scans were analysed according to the MAGNIFI-CD score, proposed by Hindryckx et al. [10], by an experienced radiologist specializing in digestive disease at baseline, 1 year and 3 years after the injection. The calculation of the score is based on the following parameters: type of fistula (simple, complex), hyper-intensity of the primary tract on post-contrast T1-weighted images, dominant feature (fibrous, inflammatory or fluid), length and extension of the fistula and presence of an inflammatory mass/collection. The MAGNIFI-CD score ranges from 0 (inactive disease) to 25 (severe disease). An improvement in the mean MAGNIFI-CD MRI score was observed at 3 years compared to baseline (17.8 ± 4.9 to 14.7 ± 4.7) (Table 3). The dominant feature of the fistula tract (fibrous, inflammatory or fluid) was the only parameter numerically improved. Finally, the number, length and extension of the fistulas did not change with treatment over time. In patients with combined remission at 3 years, the MAGNIFI-CD score improved individually in each patient and improved overall at 3 years compared to baseline (16.8 ± 5.9 to 11.5 ± 4.3) (Table 2). In contrast, in patients who failed treatment and those with recurrence, the score was similar at baseline and 3 years (19.3 ± 3.0 to 19.5 ± 4.0) (Table 3).

## Discussion

The injection of adipose tissue-derived products is emerging as a new therapeutic option. Various Preclinical models have documented the superiority of ADSVF over purified stem cells for tissue repair, lying on the heterogeneity of ADSVF that gathers various cell types displaying synergistic regenerative properties [6]. In addition, the local injection of both ADSVF and microfat is expected to provide complementary effects: ADSVF supports strong regenerative effects while microfat allows a volumizing effects that facilitates closure of fistulas [8]. We reported here that local injection of ADSVF and microfat did not cause any adverse effects over a 3-year follow-up period, providing evidence of a good long-term safety profile. The adverse events observed were considered to be expected as Crohn's disease progresses.

Importantly, this procedure led to combined remission for 70% of patients at 3 years. This high rate of efficacy provides the first evidence of a long-lasting healing effect of the innovative procedure, as 83% of patients maintained the combined remission observed at 1 year. In addition, our results also indicate a possible delayed effect of SVF and microfat. Indeed, two patients achieved combined remission only at 3 years. This hypothesis is supported by the absence of change in pharmacological treatment or surgery of the treated fistula in one patient. However, for the other patient who had a substantial treatment change and received surgical drainage with setons, it is difficult to specifically attribute the positive outcome to the study procedure. Of note, the level of remission at 3 years was not associated with a significant improvement in the PDAI score, whereas it was at 1 year. Consistently, in the ADMIRE study, success of perianal fistulas healing with expanded allogeneic MSCs did not impact the PDAI score at 24 and 52 weeks follow-up [4, 5].

Although the promise of ADSVF-based therapy has been documented in various pathologies, long-term efficacy is rarely reported. We previously reported data in favour of the long-term safety and efficacy of ADSVF injection in the hands of patients with systemic sclerosis [11]. In the field of Crohn’s disease, long-term studies only concerned expanded MSC, mainly reported data from 1 year follow-up [12] or evaluated repeated cell-based therapy procedures. Ciccocioppo and colleagues reported a 4-year follow-up with a 37% probability of fistula relapse-free survival after multiple injections of bone marrow MSCs [13]. Guadalajara et al. also described a two-step injection procedure of ADSCs mixed with fibrin glue with 40% (2/5) of patients sustaining remission at 3 years [14]. A common point between these studies was the limited number of patients followed in the long term [13, 14].

In conclusion, we confirmed the safety profile of an innovative cell-based therapy approach combining ADSVF and autologous microfat injection in refractory complex perianal fistulas of Crohn's disease and provided efficacy data in long-term assessment. As it was a pilot protocol, our study was limited by its retrospective design, the small number of patients and the absence of a control group. Accordingly, a dedicated randomized placebo-controlled trial is currently conducted (NCT04010526) to assess the efficacy of ADSVF and microfat and define the place for this approach in the current therapeutic arsenal.

## Data Availability

All data generated or analysed during this study are included in this published article.

## Abbreviations

ADICROHN: ADIpose-derived stromal vascular fraction with microfat for refractory perianal CROHN’s fistulas;
ADSVF: Adipose-derived stromal vascular fraction;
MRI: Magnetic resonance imaging
MSC: Mesenchymal stem cell
PDAI: Perianal Crohn's Disease Activity Index
SIBDQ: Short Inflammatory Bowel Disease Questionnaire
